# Facilitation of neuropathic pain by the NPY Y1 receptor-expressing subpopulation of excitatory interneurons in the dorsal horn

**DOI:** 10.1038/s41598-019-43493-z

**Published:** 2019-05-10

**Authors:** Tyler S. Nelson, Weisi Fu, Renée R. Donahue, Gregory F. Corder, Tomas Hökfelt, Ronald G. Wiley, Bradley K. Taylor

**Affiliations:** 1 Department of Anesthesiology, Center for Neuroscience at the University of Pittsburgh, Pittsburgh Center for Pain Research, and the Pittsburgh Project to end Opioid Misuse, University of Pittsburgh, Pittsburgh, PA USA; 2 Center for the Neural Basis of Cognition, University of Pittsburgh, Pittsburgh, PA USA; 3 Department of Physiology, University of Kentucky Medical Center, Lexington, KY USA; 4 Department of Psychiatry and of Neuroscience, University of Pennsylvania, Philadelphia, PA USA; 5 Department of Neuroscience, Karolinska Institutet, Stockholm, Sweden; 6 Department of Neurology, Vanderbilt University, Nashville, TN USA

**Keywords:** Chronic pain, Spine structure

## Abstract

Endogenous neuropeptide Y (NPY) exerts long-lasting spinal inhibitory control of neuropathic pain, but its mechanism of action is complicated by the expression of its receptors at multiple sites in the dorsal horn: NPY Y1 receptors (Y1Rs) on post-synaptic neurons and both Y1Rs and Y2Rs at the central terminals of primary afferents. We found that Y1R-expressing spinal neurons contain multiple markers of excitatory but not inhibitory interneurons in the rat superficial dorsal horn. To test the relevance of this spinal population to the development and/or maintenance of acute and neuropathic pain, we selectively ablated Y1R-expressing interneurons with intrathecal administration of an NPY-conjugated saporin ribosomal neurotoxin that spares the central terminals of primary afferents. NPY-saporin decreased spinal Y1R immunoreactivity but did not change the primary afferent terminal markers isolectin B4 or calcitonin-gene-related peptide immunoreactivity. In the spared nerve injury (SNI) model of neuropathic pain, NPY-saporin decreased mechanical and cold hypersensitivity, but disrupted neither normal mechanical or thermal thresholds, motor coordination, nor locomotor activity. We conclude that Y1R-expressing excitatory dorsal horn interneurons facilitate neuropathic pain hypersensitivity. Furthermore, this neuronal population remains sensitive to intrathecal NPY after nerve injury. This neuroanatomical and behavioral characterization of Y1R-expressing excitatory interneurons provides compelling evidence for the development of spinally-directed Y1R agonists to reduce chronic neuropathic pain.

## Introduction

Peripheral nerve damage can lead to a debilitating neuropathic pain syndrome that persists for years^1^. Even the most powerful opioid analgesics lack reliable efficacy, and instead cause an unacceptable set of adverse effects that often includes addiction^2^. To address this problem, distinct populations of excitatory interneurons have been identified within the spinal dorsal horn microcircuitry that are required for the behavioral expression of neuropathic pain^3–8^. These subpopulations can be defined by the expression of either a small molecule neurotransmitter (e.g. gamma-aminobutyric acid, GABA), a neuropeptide transmitter (e.g. somatostatin)^5^, transporter protein (e.g. vesicular glutamate transporter 3, VGlut3)^7^, or opioid receptor (e.g. DOR)^8^; however, none of these neural population have been found to be readily druggable targets for the development of pharmacological agents directed at non-opioid neurotransmitter receptors.

Both exogenous and endogenous NPY acts at Y1Rs within the dorsal horn to inhibit neuropathic pain. Intrathecal administration of NPY or the Y1 receptor agonist Leu^31^ Pro^34^ NPY dose-dependently reduced mechanical and cold hypersensitivity after spared sural nerve injury^9,10^ or chronic constriction injury to the sciatic nerve^11^. Likewise, either conditional NPY knockdown in NPY^tet^-transgenic mice or intrathecal administration of the Y1R antagonist BIBO 3304 elicited a robust and reliable increase in cold and mechanical hyperersensitivity^12^, indicating that neuropathic hyperalgesia is tonically inhibited by NPY that is endogenously released within the dorsal horn.

Y1Rs are highly expressed at the dorsal horn of the spinal cord, including several populations of small interneurons located throughout laminae I–III^13–17^. Immunohistochemical studies have localized Y1R to the dendrites and somas of somatostatin-positive dorsal horn neurons^18^. Since this population expresses VGLUT1 and VGLUT2^19,20^, Y1R-expressing cells have been presumed to be glutamatergic, and thus excitatory^17,21^. Indeed, we have postulated that Y1R-expressing cells are excitatory interneurons under an NPY-mediated inhibitory influence^22^; however, rigorous immunohistochemical co-labeling of Y1R cell bodies in wild-type (non-transgenic) spinal cord tissue with markers of excitatory neurons has been difficult to interpret due to the intense plexus of dendritic and terminal staining that surrounds Y1R-positive cells. To enhance Y1R resolution within dorsal horn neurons, we developed and evaluated a new intrathecal NPY injection strategy to promote receptor internalization, thereby concentrating Y1R from more distal dendritic locations to within the cell soma; the enhanced signal allowed quantification of Y1R co-localization with multiple markers of excitatory and inhibitory interneurons.

Next, to address the functional significance of spinal Y1R-expressing neurons to the development of neuropathic pain, we selectively lesioned Y1R-expressing dorsal horn interneurons using intrathecal administration of the NPY-conjugated ribosomal toxin, NPY-saporin^21–24^. NPY-saporin selectively, reliably, and dose-dependently delivers the cytotoxic ribosome inactivating protein, saporin, into Y1R neurons following NPY Y1 receptor-mediated endocytosis^21,24^. As with other peptide-saporin conjugates, NPY-saporin is selectively internalized by somatodendritic Y1 receptors on dorsal horn interneurons, while sparing axon terminals including those of Y1R-expressing DRG neurons^21^. Our approach, which uses NPY-saporin, readily discriminates between targets on cell bodies and axon terminals^21^, and avoids pitfalls associated with the use of Y1R-Cre transgenic mice that include germline recombination, transient expression, and aberrant expression at non-targeted sites^25^.

## Methods

### Animals

Male Sprague-Dawley rats (Charles Rivers Laboratories), delivered at 165–205 g and weighing 235–260 g at time of surgery were used throughout the study. Animals were housed in a temperature-controlled room on a 12-hr light/dark cycle and were given chow and water *ad libitum*. All animal use protocols were approved by the IACUC of the University of Kentucky. All experiments and methods were performed in accordance with institutional relevant guidelines and regulations and in accordance with the NIH Guide for the Care and Use of Laboratory Animals.

### Intrathecal delivery of NPY and NPY-saporin

#### NPY

Our initial attempts to visualize Y1R cell bodies failed due to the high background staining from the dense plexus of central terminals of primary afferent neurons, as noted previously^15^. To overcome this problem, Brumovsky *et al*. used tyramide signal amplification (TSA) method combined with confocal imaging in thin optical sections to visualize Y1R-expressing cell bodies^15^. Here, we used an alternative approach to enhance visualization of Y1R interneurons: we delivered spinal NPY to promote receptor internalization, thereby concentrating Y1R from more distal dendritic locations to within the cell soma. This was achieved following two intrathecal injections of NPY (30 µg) separated by 1 hour. One hour after the second injection, rats were deeply anesthetized with isoflurane and perfused with 0.1 M PBS containing heparin (10,000 USP units/L) followed by 10% buffered formalin.

#### NPY-Saporin

To selectively ablate spinal Y1R-expressing interneurons, the saporin-conjugated peptide NPY-saporin, or a control blank-saporin (Advanced Targeting Systems, San Diego, CA) was intrathecally injected at the lumbar level in a volume of 10 μl using a 25 μl Hamilton microsyringe attached to a 27-gauge disposable sterile needle under isoflurane anesthesia (5% induction; 1.5–2% maintenance). Blank saporin is an 11-amino acid, randomly-mixed version of the sequence of melanocyte-stimulating hormone. Its amino acid residues are typical of peptides that bind to G-protein-coupled receptors although this random peptide has no homologous sequences that which it can bind to *in vivo*. Blank-saporin was delivered at a 1000 ng dilution. The needle was inserted into the subarachnoid space through the intervertebral foramen. A tail flick response was used as verification of correct placement of the needle and successful saporin delivery was verified via decreases in Y1R immunoreactivity.

### Spared nerve injury (SNI) surgery

Fourteen days after intrathecal NPY-saporin or blank-saporin injection, animals underwent SNI surgery. SNI was performed as described previously^9,26^. Anesthesia was induced and maintained with 5% and 2–3% isoflurane, respectively. After shaving and Betadine wipe of the left hind limb, an incision was made in the skin at the level of the trifurcation of the left sciatic nerve. The overlying biceps femoris muscles were retracted, exposing the tibial, common peroneal, and sural nerve branches. The common peroneal and tibial nerves were ligated with 6-0 silk (Ethicon, Somerville, NJ), and then the knot and adjacent nerve (2 mm) were transected, leaving the sural branch intact. The muscle was sutured with 4-0 silk sutures and the wound was closed with 9-mm metal clips, followed by Neosporin®.

### Behavioral testing

Behavioral testing was conducted at baseline and 3, 8, 11, 14, 17, 21, 28, 35, 42, and 54 days post-SNI. In a separate cohort of rats, spinal cord tissue and dorsal root ganglia tissue were collected at 14 days post-SNI surgery for immunohistochemistry. For naïve studies, rats received rotarod training for 2 days, then received intrathecal injection of NPY-saporin or blank-saporin. Testing in these rats was completed 2–3 weeks post intrathecal injection.

#### Mechanical Hyperalgesia, von Frey

To evaluate sensitivity to a non-noxious mechanical stimulus, we used an incremental series of 8 von Frey filaments of logarithmic stiffness (0.4–15 grams). The 50% withdrawal threshold was determined using the Up-Down method^27^. Each filament was applied perpendicular to the lateral hindpaw surface with sufficient force to cause a slight bending of the filament. A positive response was defined by a rapid withdrawal of the paw within 5 seconds.

#### Mechanical Hyperalgesia, noxious pin

To evaluate sensitivity to a noxious mechanical stimulus, we gently and rapidly applied the point of a dull pin to the lateral aspect of the hind paw, avoiding damage to the skin. The duration of time with which the animal raised this paw was recorded, with a cut-off of 30 seconds. Three measurements were averaged.

#### Mechanical hyperalgesia, paw pressure

To assess sensitivity to increasing noxious mechanical pressure, animals were lightly restrained while extending the hind paw. The plantar surface was placed on the plinth between the calipers of the Randall-Selitto device (IITC) and increasing gram force was gradually applied on the surface of the paw until the animal exhibited a withdrawal or vocalization. Three measurements of gram force at time of response were averaged.

#### Cold Allodynia, acetone drop

To evaluate the response to a cool stimulus, we used a piece of PE-90 tubing, melted at the tip, to apply a drop (10–12 ul) of acetone to the lateral aspect of the ventral hindpaw. The duration of time with which the animal raised this paw was recorded, with a cut-off of 30 sec. Three measurements were averaged.

#### Heat Hyperalgesia, Hargreaves’s irradiant heat

To evaluate the response to a heat stimulus, rats were placed in a clear Plexiglas box on a glass floor. The thermal stimulus consisted of a radiant heat source positioned under the glass floor directly beneath the hind paw. Voltage intensity was adjusted such that paw withdrawal latency was 10 ± 0.5 seconds. If the animal did not respond within 20 seconds, the heat was discontinued to prevent damage to the paw. Withdrawal responses to three stimulus pairs, delivered every five minutes, were averaged.

#### Heat hyperalgesia, hot plate

To evaluate the response to a heated floor stimulus, a single rat was placed on a 48, 52, or 56 °C surface within an acrylic enclosure (Columbus Instruments). The animal was immediately removed from the enclosure when it jumped, licked, or lifted a hind paw. Response latencies to three trials were averaged.

#### Motor coordination, rotarod

To evaluate motor coordination, rotarod testing was performed at 7 and 14 days post-saporin injection. Rats were trained on two training days prior to intrathecal injection. The rotarod accelerated 0.5 rpm every 5 sec, with a maximum speed of 40 rpm. Training involved repeated placement on the rotarod until one of the following was achieved: exposure to 20 sessions, or successful performance of at least 150 seconds for three consecutive trials. For experimental testing, rats were placed on the rotarod at one and two weeks post-intrathecal injection. Performance time, recorded in seconds, was determined when the animal fell off of the rotating bar, thus breaking a light beam. Five consecutive trials were averaged for each animal.

#### Integrated Exploratory Activity

To evaluate innate exploratory behaviors of rats in a dark novel “open field”, a single rat was placed in a clear Plexiglas box on a Plexiglas floor. We evaluated activity using the automated Photobeam Activity System (PAS) with Flexfield Animal Activity System (San Diego Instruments, Inc, San Diego), coupled to a computer to eliminate human interaction and bias. Using 32 infrared photobeams, six main parameters were measured in six 5−min intervals: rearing events, active time and resting time, beam breaks, and distance traveled.

### Immunohistochemistry

Animals were deeply anesthetized with pentobarbital (Fatal Plus, diluted to 200 mg/kg i.p., Med-Vet International, Mettawa, IL) and perfused transcardially with 200 ml of room temperature (RT), 0.01 M phosphate buffered saline (PBS) with heparin (10,000 USP units/L) followed by 300 ml of ice-cold fixative (10% buffered formalin). The cord was removed and post-fixed for 4 hr in 10% buffered formalin (4 °C) and then cryoprotected (30% sucrose in 0.01 M PBS for 36–96 hr). L4-L6 transverse sections (35–40 μm) were cut on a freezing microtome and collected in 0.01 M PBS. The sections were washed three times in 0.01 M PBS and then pretreated with 3% normal goat or donkey serum serum and 0.3% Triton X-100 to block non-specific binding. Sections were then incubated in a primary antibody, rabbit anti-Fos (1:20,000, Calbiochem), rabbit anti-Y1R (1:5,000, courtesy of Janice Urban), rabbit Anti-CGRP for mouse/rat (1:20,000, Bachem) and rabbit anti-NK1R (1:10,000, Neuromics) overnight at RT on a slow rocker. For fluorescence co-labeling studies, we used a rabbit anti-Y1R antibody (Neuromics) derived from the same antigen as that developed by Janice Urban and colleagues, but at a more concentrated dilution (1:500). Spinal cord sections were then co-incubated with Y1R antibody together with either markers of excitatory interneurons (mouse anti-calbindin, 1:1,000, Sigma; goat anti-calretinin, 1:5,000, Swant; guinea pig anti-PKCγ, 1:10,000, courtesy of Allan Basbaum; mouse anti-somatostatin, 1:500, GeneTex) or a marker of inhibitory interneurons (goat anti-Pax-2, 1:1,000, R&D systems). The tissue was then washed three times in 0.01 M PBS, and incubated at RT in secondary antibody for either enzyme (1:200 dilution) or fluorescent (1:700 dilution) labeling. Secondary antibodies used for the co-localization studies were: Alexa 568-conjugated goat anti-rabbit; Alexa 568-conjugated donkey anti-rabbit; Alexa 488-conjugated goat anti-mouse; Alexa 488-conjugated goat anti-guinea pig; Alexa 488-conjugated donkey anti-goat (all 1:1,000; Invitrogen). For fluorescent IB4 labeling, tissue was incubated in a primary IB4 antibody conjugated to FITC (1:500, Sigma) and cover-slipped with Prolong Gold with DAPI mounting medium (Molecular Probes).

### Confocal microscopy, image processing, and quantification of Y1R co-localization with INs

Representative confocal images of Y1R co-labeling with markers of excitatory or inhibitory interneurons were acquired with a Leica ABOS TCS SP5 inverted laser scanning confocal microscope, fitted with a 100x oil immersion objective (numerical aperture 1.46). The microscope is a Leica DMI 6000 with LAS AF 2.7.2.9586 software. Laser excitation lines and emission windows for the different fluorophores were: Alexa Fluor 488 - excitation 488 nm (Ar laser), emission 505–555 nm; Alexa Fluor 568 - excitation 543 nm (diode laser), emission 565–615 nm; DAPI - excitation 405 nm (HeNe laser), emission 435–485 nm. Line averaging was used to decrease signal to noise ratio. Adobe Illustrator CS6 (Adobe Systems Inc., Mountain View, CA) was used to assemble the multi-panel figures.

Quantification of staining in randomly-selected sections was performed with NIS Elements Advanced Research software. To distinguish immunohistochemical staining patterns of tibial and sural terminals, we selected ROIs spanning the medial-central and central-lateral regions, respectively, of lamina II of the tibial and sural across the mediolateral axis of the dorsal horn. Only DAPI-labeled cells were counted. Three animals per group and three slices per animal were quantified for Y1R and/or calbindin, calretinin, PKCγ, or Pax-2.

### Fluorescence microscopy, image processing, and quantification of Fos immunohistochemistry

Digital photomicrographs were captured from lumbar segment L4-L5 with a Nikon TE2000-E microscope with Metamorph software (Version 6.1r4, Universal Imaging Corp.). Immunoreactivity was quantified with NIH custom ImageJ software. Integrated density was determined by thresholding the images using the default algorithm within ImageJ to reduce background and include positively stained cells in spinal cord dorsal horns from the L4-L5 lumbar region. Integrated density of the region of interest (ROI) is equal to the product of ROI area and mean gray value. The mean gray value represents the sum of the intensity values for all pixels above the threshold in the ROI divided by the number of pixels above threshold within the ROI. This method controls for differences in background between slices and subjects. For quantification of Fos, an observer blinded to treatment manually counted punctate immunoreactive profiles in lamina I-V. Six animals per group and 4–6 slices per animal were quantified for Y1R, CGRP, IB4, or NK1R.

### Statistical analysis

Data were analyzed using Prism software (GraphPad, San Diego CA). All data are expressed as mean ± SEM. Statistical significance was set at P < 0.05. Behavioral data were analyzed by two-way ANOVA with treatment as the between-subjects factor and time as the within-subject factor. Other data were analyzed by one-way or two-way ANOVA and Bonferroni or Tukey’s post-hoc tests, or unpaired, two-tailed student T-tests.

## Results

### Expression of Y1R with markers of excitatory but not inhibitory interneurons in dorsal horn

**Figure 1:**
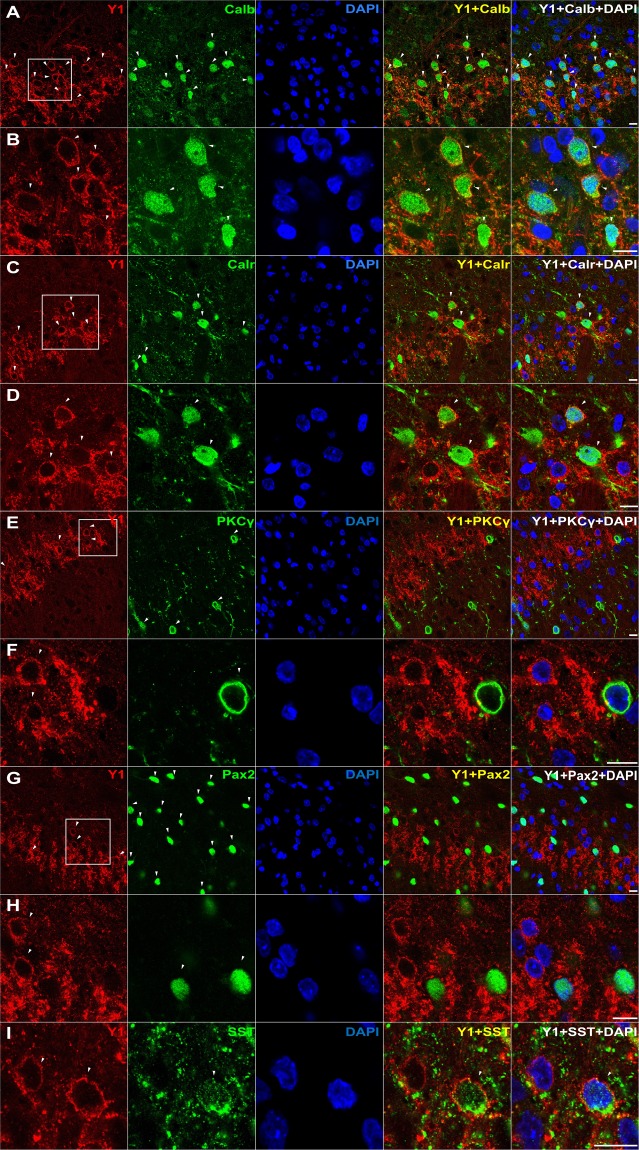
Y1R-expressing profiles often co-express calbindin, calretinin, and/or somatostatin, but neither PKCγ nor Pax2. Co-staining of Y1R with DAPI and antibodies against (**A**,**B**) calbindin, (**C**,**D**) calretinin, (**E**,**F**) PKCγ, (**G**,**H**) Pax2, or (**I**) somatostatin. Confocal images of transverse L4-L6 sections were taken with a 100X objective from lamina II. Dorsal side is up. Images in B, D, F, and H are zoomed in from the white square boxes shown in A, C, E, and G, respectively. Arrows indicate instances of Y1R colabeling. Scale bars: 10 µm.

**Figure 2:**
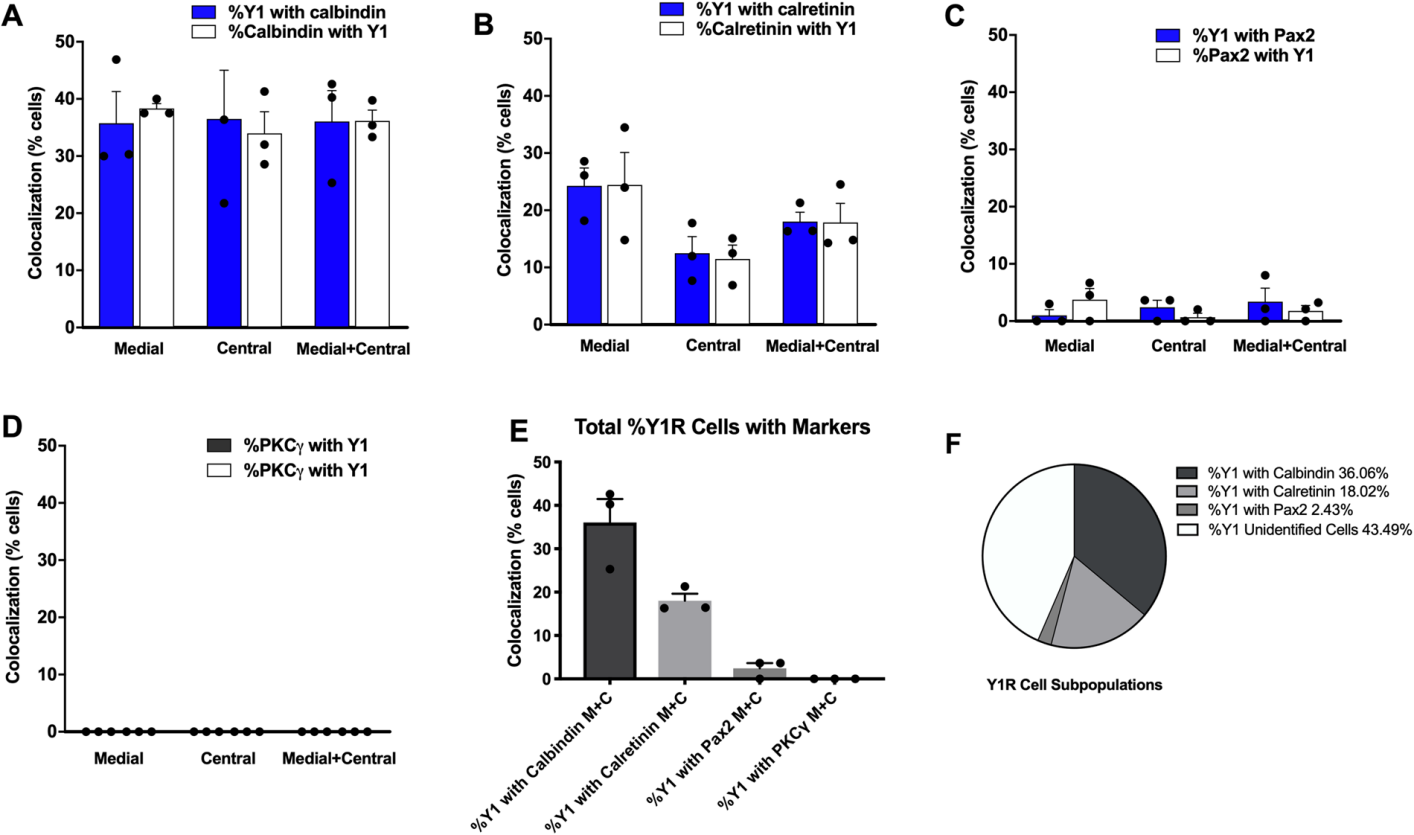
Quantification of Y1R co-localization. Quantification of colocalization in medial and central dorsal horn of (**A**) Calbindin-ir, (**B**) Calretenin-ir, (**C**) Pax2-ir, and (**D**) PKCγ-ir with Y1R-ir. The relative distribution of subpopulations of Y1R-expressing neurons as determined by % colocalizations can be seen in (**E**,**F**). N = 3 rats per antibody with N = 3 transverse sections averaged per animal.

Y1R immunoreactivity (Y1R-ir) in the dorsal horn presents as a dense plexus of axons and dendrites that complicates analysis of co-labeling. We reduced this problem by pretreating animals with two intrathecal injections of NPY (30 µg) separated by 1 hour so as to promote receptor internalization, thereby concentrating Y1R from more distal dendritic locations to within the cell soma. As illustrated in Fig. 1, NPY pretreatment concentrated Y1R-ir within the cell soma, thereby permitting a more accurate assessment of spinal Y1R-expressing populations. We found Y1R-ir to co-localize with multiple markers of excitatory neurons in superficial lamina: calbindin (Figs 1A,B and 2A), calretinin (Figs 1C,D and 2B), and somatostatin (Fig. 1I), but neither PKCγ-ir (which labels a band in inner lamina II) (Figs 1E,F and 2D) nor Pax2-ir (a marker of inhibitory neurons) (Figs 1G,H and 2C).

The colocalization of Y1R and somatostatin is consistent with studies utilizing Y1R-ir^18^ and transcriptional profile analyses^28^. Neuronal phenotypes can be classified based upon the transcription factors that regulate the development of their lineage^29–31^. Notable is Pax2, which continues to be expressed in mature GABAergic neurons. We found very little co-localization with Pax2 (Fig. 2C), and therefore our results indicate that Y1R-expressing cells represent a large subpopulation of excitatory but not inhibitory interneurons.

### NPY-saporin selectively ablated Y1R-expressing spinal interneurons

**Figure 3:**
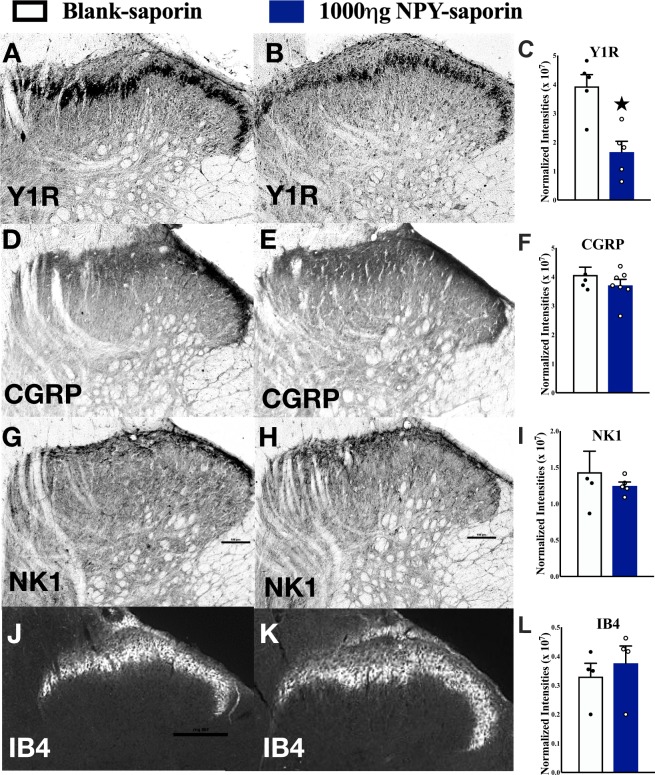
NPY-saporin lesion selectively reduces Y1R-ir in the dorsal horn. Y1R, CGRP, NK1, or IB4 immunostaining in the dorsal horn of naïve rats 14 days after intrathecal injection of (**A**,**D**,**G**,**J***)* blank saporin or (**B**,**E**,**H**,**K**) 1000 ng NPY-saporin. NPY-saporin decreased (**C**) Y1R but did not change immunostaining in the dorsal horn of (**F**) CGRP, (**I**) NK1-R or (**L**) IB4 as compared to blank-saporin controls. Values are expressed as integrated density of staining in dorsal horn. ★P < 0.05 compared to blank-saporin. Data represent mean ± SEM. Scale bars: 100 µm.

The highest dose of NPY-saporin in Wiley *et al*. (2009) was 750 ng. This dose reduced Y1R immunoreactivity in the dorsal horn by approximately 40% (see their Table 1)^21^. In an attempt to lesion a greater number of Y1R-expressing neurons, we used not only the 750 ng dose, but also a higher dose of 1000 ng. As illustrated in Fig. 3(A–C), we found that the 1000 ng dose reduced Y1R staining by approximately 50% as compared to control treatment with an injection of a scrambled peptide conjugated to the saporin toxin. To determine the selectivity of intrathecal NPY-saporin for Y1R spinal interneurons, we evaluated not only Y1R-ir, but also NK1R (a marker of spinal cord nociception-responsive projection neurons that ascend to the brain), as well as CGRP and IB4 (markers of the central terminals of primary afferent terminals). By contrast, NPY-saporin did not change NK1R-ir, IB4-ir or CGRP-ir relative to the blank-saporin (P > 0.05; Fig. 3D–L). These findings indicate that Y1R-expressing primary afferents and projection neurons were spared by the toxin, and are consistent with Wiley and colleagues who reported that intrathecal NPY-saporin did not change the number of Y1R-expressing DRG neurons, nor dorsal horn staining for either NK-1R or mu opiate receptor (MOR) as compared to blank-saporin^21^.

### NPY-saporin reduced the development and maintenance of neuropathic pain without changing normal motor behaviors or nociception

**Figure 4:**
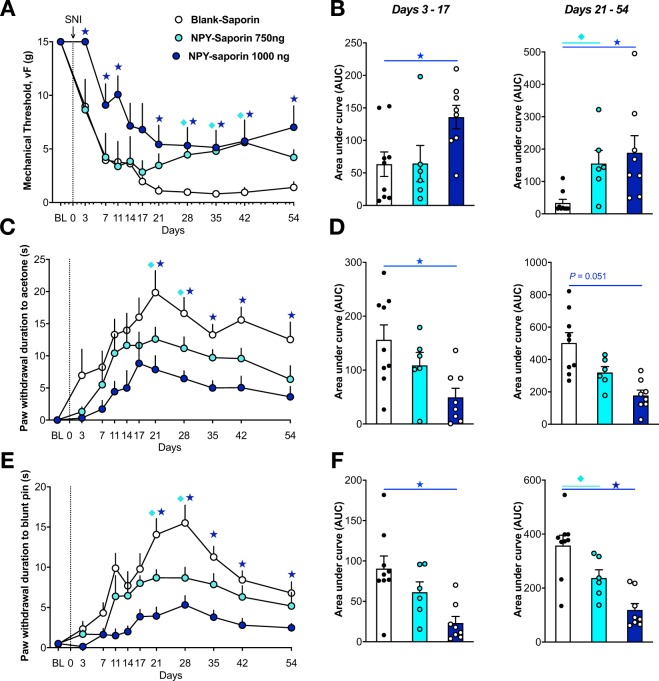
Lesion of Y1R-expressing spinal neurons reduces the severity of neuropathic pain. (**A**,**C**,**E**) Intrathecal NPY-saporin (750 ng or 1,000 ng) reduced the development of (**A**) mechanical hypersensitivity to von Frey filaments (F_2,20_ = 5.516, *P* = 0.01), (**C**) cold response duration during acetone evaporation (F_2,20_ = 9.889, *P* = 0.001), and (**E**) mechanical response duration to blunt pin (F_2,20_ = 13.43, *P* = 0.0002), compared to intrathecal Blank-saporin. Repeated Measures Two-way ANOVA + Bonferonni. (**B**,**D**,**F**) Area Under the Curve (AUC) analyses for days 3–17 (*P* < 0.05 for all panels) and 21–54, respectively. One-way ANOVA + Bonferonni. N = 6–9 per group. ★P < 0.05 compared to Blank-saporin. Dots represent individual subjects within the analysis. Data represent mean ± SEM.

Previous studies indicate that NPY-saporin decreased behavioral signs of early inflammatory pain^24^, including Phase II of the formalin test, and reduced hotplate reflex responses to low (44 °C) intensity heat^21^. To determine the contribution of Y1R-expressing dorsal horn neurons to chronic neuropathic pain, we delivered NPY-saporin 14 days prior to SNI and evaluated the progression of nerve-injury induced hyperalgesia and allodynia over several weeks. SNI produced mechanical hypersensitivity (von Frey), cold hypersensitivity (acetone drop), and mechanical hyperalgesia (blunt pin prick) that peaked at approximately 21 days in blank-saporin-treated control rats (Fig. 4). While both mechanical and cold hypersensitivities reached a steady-state that was maintained until at least 54 days post-SNI, pin prick mechanical hyperalgesia gradually decreased from Day 28 through Day 54. Relative to rats treated with blank-saporin, NPY-saporin dose-dependently reduced mechanical hypersensitivity (Treatment: F_2,20_ = 6.42, P = 0.007), cold hypersensitivity (Treatment: F_2,20_ = 9.76, P = 0.0011), and mechanical hyperalgesia (Treatment: F_2, 20_ = 13.43, P = 0.0002). Secondary analysis of each NPY-saporin group compared to the blank-saporin control group revealed that the 750 ng dose did not change behavioral signs of neuropathic pain at earlier (*Days 3–17)* timepoints (P > 0.05), but decreased vF mechanical hypersensitivity (F_1,13_ = 12.0, P = 0.0042), cold hypersensitivity (F_1,13_ = 4.53, P = 0.05), and mechanical hyperalgesia (F_1, 13_ = 5.29, P = 0.039) at later timepoints (*Days 21–54)*. By contrast, the 1000 ng dose of NPY-saporin decreased behavioral signs of neuropathic pain at both earlier timepoints (*Days 3–17)*: vF mechanical hypersensitivity (F_1,15_ = 7.93, P = 0.013), cold hypersensitivity (F_1,15_ = 8.20, P = 0.012), pin prick mechanical hyperalgesia (F_1, 15_ = 14.0, P = 0.002), as well as later timepoints (*Days 21–54)*: vF mechanical hypersensitivity (F_1,15_ = 14.2, P = 0.0019), cold hypersensitivity (F_1,15_ = 18.0, P = 0.0007) and mechanical hyperalgesia (F_1,13_ = 25.4, P = 0.0001).

Analysis of area under the curve (AUC) illustrates that effect size depends on the somatosensory modality, time of testing after injury, and dose of NPY-saporin, Thus, while the 750 ng dose did not change mechanical withdrawal thresholds as compared to blank-saporin controls over Days 3–17 (just a 1.6% increase), it produced a quite robust, greater than 3-fold increase in mechanical thresholds over Days 21–54 (360% increase); also the 750 ng dose decreased behavioral signs of noxious mechanical hyperalgesia and cold allodynia by approximately one-third regardless of timepoint (Days 3–17: 30.3% reduction in cold withdrawal response, 32.3% increase in pin prick withdrawal; Days 21–54: 36.3% reduction in cold withdrawal response, 33.7% increase in pin prick withdrawal). The 1000 ng dose produced even greater effects as compared to blank-saporin controls over Days 3–17 (increased mechanical withdrawal thresholds by 114%, reduced cold withdrawal response by 68.5%, and increased pin prick withdrawal by 74.4%) and Days 21–54 (increased mechanical withdrawal thresholds by 458%, reduced cold withdrawal response by 64.6%, and increased pin prick withdrawal by 66.8%). In summary, the effects of NPY-saporin on neuropathic pain behaviors are strongest at later timepoints, and the higher dose recruits an additional effect on mechanical allodynia at early timepoints.

**Figure 5:**
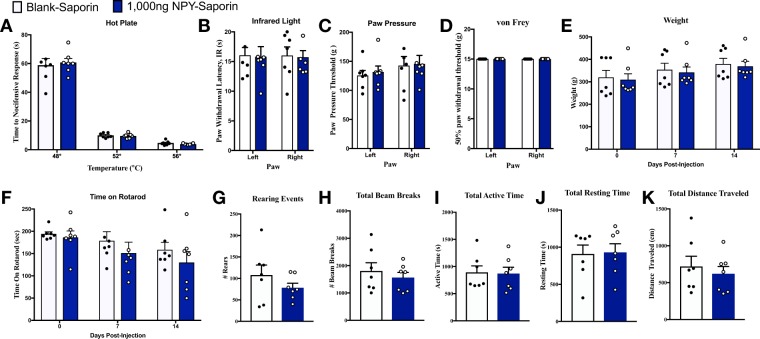
Lesion of Y1R-expressing spinal neurons does not alter basal nociception or motor control. The effect of intrathecal blank-saporin or NPY-saporin (1000 ng) on rat nociceptive behavior in the (**A**) hotplate assay and (**B**) Hargreave’s thermal assay for heat hyperalgesia, and the (**C**) Randel-Siletto paw pressure assay and the (**D**) von Frey filament assay for mechanical sensitivity. (**E**) Neither blank-saporin nor NPY-saporin changed body weight. The effect of intrathecal blank-saporin or NPY-saporin (1000 ng) on rat motor behavior in the (**F**) Rotarod test for motor coordination, and (**G**–**K**) general activity measures in an open field arena. Dots represent individual subjects within the analysis. Data represent mean ± SEM.

We evaluated the effect of NPY-saporin on numerous parameters of acute pain and motor control (Fig. 5). As compared to blank-saporin, NPY-saporin did not change thermal or mechanical sensitivity, body weight, motor coordination, ambulatory behavior, nor exploratory behaviors in an open field activity box (P > 0.05). This data suggests that the anti-hyperalgesia effects of NPY observed in Fig. 4 do not apply to all modalities of acute nociception, and are not confounded by motor side effects. Our results indicate that NPY-saporin did not change hindpaw withdrawal thresholds in response to application of noxious heat at 48 °C, 52 °C, or 56 °C. This is consistent with Wiley *et al*. (2009) who reported no effect in response to 47 °C or 52 °C (antinociceptive effects were observed only at a much lower temperature of 44 °C)^21^.

### Nerve injury does not decrease NPY-Y1R signaling in the dorsal horn

Peripheral nerve injury decreases the expression of several neuropeptide transmitters and receptors in dorsal root ganglion and spinal cord^27,32^, as well as the signal transduction of pain inhibitory GPCRs in in the brain^33^. For example, injury-induced decreases in the dorsal horn expression of the mu opioid receptor is accompanied by decreases in capacity for opioid-induced analgesia^34,35^, a mechanism that might explain the poor efficacy of opioid analgesics for neuropathic pain^36^. Whether nerve injury produces similar changes in Y1 signaling and responsiveness to NPY antinociception is unknown. To address this question, we evaluated Y1R density and NPY-induced FOS activation of Y1R-expressing neurons after SNI. We used a polyclonal antibody whose Y1R specificity in rat tissue was confirmed using western analysis, preadsorption of the antibody with peptide, and preimmune serum controls^37^.

**Figure 6:**
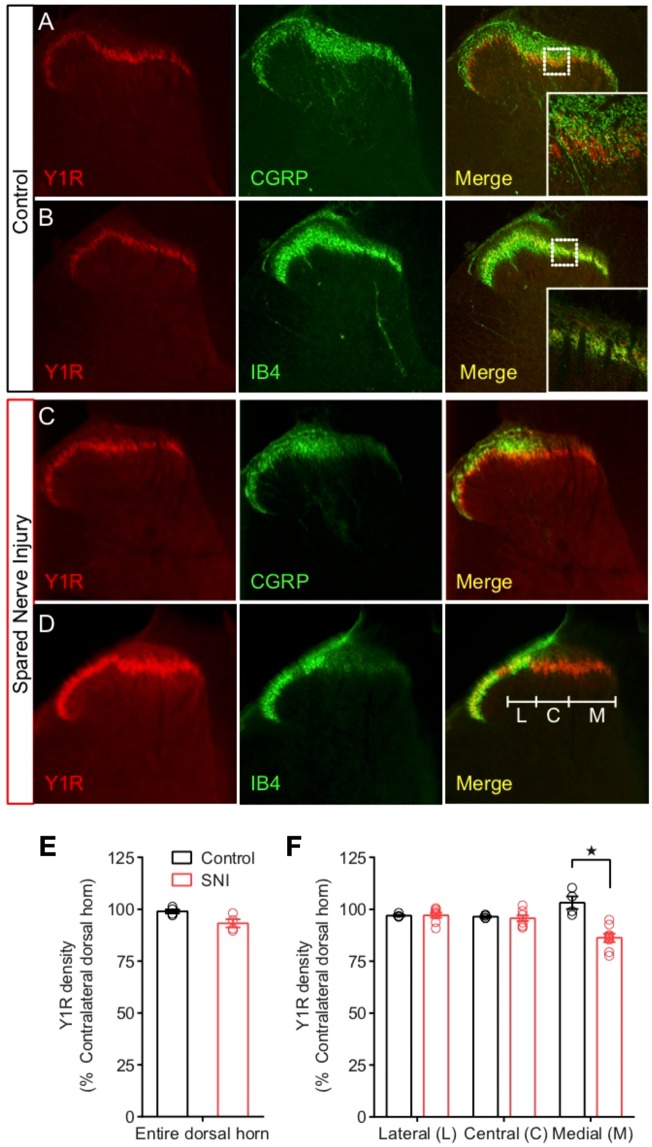
Peripheral nerve injury largely spares Y1R expression in the dorsal horn. (**A**,**B**) In uninjured rats, Y1R immunostaining of lumbar spinal cord slices shows minimal colocalization with the afferent terminal markers (**A**) CGRP and (**B**) IB4. SNI largely spared Y1R in contrast to large decreases in (**C**) CGRP and (**D**) IB4. (**E**) Quantification of Y1R immunostaining across the entire mediolateral axis of the dorsal horn revealed no significant overall loss of Y1R. (**F**) Segregation of the dorsal horn by the innervation zones of the tibial (medial, M), common peroneal (central, C) or sural (lateral, L) branches of the sciatic nerve revealed a slight decrease in Y1R density in the tibial innervation zone relative to controls. Control, N = 4; SNI, N = 8. ★P < 0.05 compared to control. Data represent mean ± SEM. Dots represent individual subjects in the analysis.

As previously described^15^, we observed a pattern of intense Y1R-ir in lamina I-II comprised of tightly-packed cell bodies, embedded in Y1R-expressing processes, surrounded by CGRP-ir nerve endings (Fig. 6A), and overlapping with IB4-positive cells (Fig. 6B). As expected, SNI substantially reduced CGRP and IB4 immunoreactivity within the innervation territories of the tibial and common peroneal nerves (Fig. 6C,D). By contrast, SNI only slightly reduced spinal Y1R-ir density within just the tibial and not the common peroneal innervation territory (Fig. 6E,F). While this slight immunoreactive reduction likely represents loss of Y1R expressed on the central terminals of primary afferent neurons that terminate in the IB4 positive laminar band, the small effect here is consistent with preliminary reports suggesting that “light” or “medium” chronic constriction injuries impact 80% or less of the sciatic nerve and, thus, would not substantially reduce spinal Y1R-ir density^16^.

**Figure 7:**
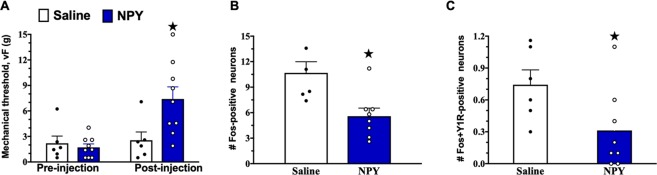
NPY reduces neuropathic mechanical hypersensitivity and light touch-evoked c-Fos expression in Y1R^+^ dorsal horn neurons. (**A**) von Frey thresholds 14 days after SNI before (pre-injection) and 60 min after intrathecal injection of saline or NPY (post-injection). Non-noxious mechanical stimulus-evoked expression of Fos-immunoreactivity in lamina I-II (**B**) for all neurons and (**C**) in Y1R-immunoreactive neurons. N = 6–9. ★P < 0.05 compared to control. Data represent mean ± SEM. Dots represent individual subjects in the analysis.

We next evaluated the effect of NPY on neuronal activity in Y1R spinal interneurons during neuropathic conditions. After intrathecal administration of NPY, we quantified the co-expression of Fos and Y1R in dorsal horn neurons. We found that intrathecal NPY increased von Frey thresholds (Fig. 7A, P < 0.05) and reduced the number of Fos-expressing cells in the dorsal horn (Fig. 7B, P < 0.01). Importantly, NPY decreased Fos expression within Y1R-expressing neurons (Fig. 7C, P < 0.05). In summary, peripheral nerve injury spares spinal Y1R density and responsiveness of Y1R neurons to NPY. These studies indicate that the Y1R retains the capacity to inhibit spinal pain transmission after nerve injury.

## Discussion

The key finding of the present studies is that selective ablation of Y1R-expressing neurons in the dorsal horn delayed the onset and reduced the intensity of behavioral signs of neuropathic hyperalgesia after peripheral nerve injury. The effect was broad spectrum, impinging upon multiple somatosensory modalities including non-noxious mechanical, noxious mechanical, and cold hypersensitivity. These results further support spinal Y1R as a potential target for the pharmacological treatment of chronic pain.

### Y1R-expressing spinal excitatory interneurons contribute to neuropathic pain

Calbindin and calretinin are primarily located in different sub-populations of dorsal horn neurons and are largely (but not exclusively) restricted to glutamatergic cells^38–40^. For the first time, we show that both are contained in Y1R-immunoreactive neurons. PKCγ-ir neurons are present throughout laminae I–III^41,42^, particularly in the inner half of lamina II, where their dendrites form a dense plexus^43,44^. Although PKCγ-ir cell bodies make numerous contacts with NPY-positive boutons and dendrites^45^ and thus might appear to be a candidate for co-expression with Y1R, this population is largely distinct from the calbindin and calretinin populations and did not colocalize with Y1R in the current study. We conclude that the vast majority of Y1R-expressing cells are excitatory interneurons that lie dorsal to the inner lamina II band that is demarcated by PKCγ staining.

We found that Y1R-ir neurons in lamina II typically contain somatostatin^18^. Most somatostatin-containing boutons contain the vesicular glutamate transporter 2, VGLUT2^19^, and somatostatin lineage-TdTomato cells extensively co-label with *Vglut2* mRNA^5^. Because excitatory interneurons and their boutons in the dorsal horn express VGLUT2^19,46,47^, it is highly likely that somatostatin-expressing neurons are excitatory. Furthermore, selective ablation of somatostatin lineage neurons, using an intersectional genetic strategy, decreased mechanical allodynia associated with SNI in the mouse, suggesting that spinal somatostatin-expressing excitatory interneurons transmit neuropathic mechanical information^5^. This is consistent with the present results showing that deletion of Y1R neurons, and thus a subset of somatostatin neurons, decreased the neuropathic allodynia in the rat.

In addition to novel immunohistochemical profiling of neuropeptide and neurotransmitter neuron populations of the dorsal horn, significant progress has been made in the past decade in understanding the development of dorsal horn spinal neuron lineages. Of note is the uncovering of specific transcription factors that determine the excitatory (glutamatergic) or inhibitory (GABAergic/glycinergic) cell fate of spinal dorsal horn neurons^48–50^. In support of our studies, *Npy1r* gene expression is almost exclusively found in Tlx3^+^ glutamatergic neurons, and rarely found in Pax2^+^ GABAergic neurons^51^. Further, transcriptomic profiling analysis has determined that *Npy1r* mRNA is significantly enriched in the somatostatin dorsal horn neuron population^28^. Single-nucleus RNA sequencing has clustered *Npy1r* into a dorsal excitatory peptidergic neuron cluster^52^, and single-cell RNA sequencing has classified *Npy1r* neurons as excitatory glutamatergic interneurons^53^.

### Y1R interneurons contribute to the development and maintenance of neuropathic pain

The effect of NPY-saporin on the development (early timepoints) and maintenance (late timepoints) of allodynia and hyperalgesia varied with dose and somatosensory modality. For example, the 750 ng dose did not change von Frey mechanical threshold at earlier timepoints, but exerted a robust increase in threshold at later timepoints. By contrast, the 1000 ng dose of NPY-saporin reduced mechanical thresholds (and cold and pinprick responses) at all timepoints. The additional efficacy of the higher NPY-saporin dose at earlier timepoints suggest that two subpopulations of Y1R-expressing interneurons differentially control the early development and the long-term maintenance of nerve injury-induced mechanical allodynia. Thus, in addition to a Y1R-expressing subpopulation that maintains neuropathic pain and is vulnerable to 750 ng NPY-saporin, there is an additional subpopulation that drives the development of neuropathic pain and is only vulnerable to the 1000 ng dose.

These studies are the first to implicate Y1Rs and Y1R-expressing dorsal horn neurons in the development of neuropathic pain and are consistent with previous studies indicating that Y1Rs contribute to the maintenance of neuropathic pain. For example, intrathecal administration of neuropeptide Y or the selective Y1R agonist Leu^31^ Pro^34^ dose-dependently reversed established markers of neuropathic pain including hyperalgesia and stimulus-evoked Fos expression in the dorsal horn^9,11^. Further studies are needed to determine whether ablation or inhibition of Y1R-expressing neurons, with either intrathecal administration of NPY-saporin or optogenetic or chemogenetic inhibition of Y1R-expressing neurons utilizing cre driver lines, will inhibit established signs of neuropathic pain when administered days to weeks after peripheral nerve injury.

### NPY-saporin selectively targets Y1R interneurons rather than central terminals of primary afferent neurons or spinal projection neurons

Y1Rs are expressed on small- to medium-sized DRG neurons and spinal cord neurons^14,54,55^. Since intrathecal NPY-saporin could conceivably cross the fibrous sheath that encases the DRG (as observed with GFP-conjugated viral particles), or be taken up by terminals of Y1R-expressing primary afferents, and attack peripheral Y1R-containing cells, one might predict that intrathecal NPY-saporin would kill not only Y1R-containing dorsal horn neurons but also DRG neurons and their unmyelinated afferents that terminate in lamina I/outer part of lamina II (CGRP-containing) or in the inner part of lamina II (isolectin B4-containing). This is unlikely for several reasons. First, the current studies indicate that NPY-saporin did not change CGRP and IB4 staining in the dorsal horn. Second, spinal Y1R does not readily co-stain with CGRP, SP, or IB4^14,55^ and is unchanged following dorsal rhizotomy^54^, indicating that its existence on the central terminals of primary afferents is sparse at best. Third, Wiley and colleagues reported that intrathecal NPY-saporin had no effect on Y1R cell counts in DRG of the fourth lumbar spinal segment^21^, indicating insufficient penetration into the DRG. Lastly, the vast majority of NPY Y2 receptors in the dorsal horn are found on the central terminals of primary afferents^56^, but a recent single-cell RNA sequencing analysis suggests the existence of Y2 receptors on a few interneurons^53^. We cannot exclude that these very few Y2R-expressing neurons were affected by NPY-saporin but consider it highly unlikely that this would affect the conclusions drawn in our study, as has been previously concluded^21^. Therefore, we conclude that the vast majority of Y1R immunoreactivity is located on dorsal horn neurons, and that NPY-saporin selectively ablates this Y1R-containing population of neurons, rather than Y1R- or Y2R-positive terminals of primary afferent neurons.

Similar lack of effect on DRG neurons has been reported after intrathecal injection of dermorphin-saporin, a mu opiate receptor-specific toxin^57^. In that study, dermorphin-saporin injections destroyed lamina II MOR-expressing interneurons but had no effect on MOR-expressing DRG neurons. Although immunotoxins, such as OX7-saporin, 192-saporin and anti-dopamine beta-hydroxylase saporin are effective suicide transport agents (killing target neurons after selective uptake into axon terminals, followed by retrograde axonal transport to cell bodies where saporin acts to induce cell death), there is considerable evidence that intrathecal neuropeptide-saporin conjugates do not affect DRG neurons^21,57,58^. Thus, there is a key difference between immunotoxins which are effective suicide transport agents and neuropeptide-toxin conjugates which are not.

Y1R-ir cells exhibited retrograde labeling in lamina I, V, and X after choleratoxin B injection at the level of the 9^th^ thoracic segment, and therefore project, at least, to the lower thoracic levels^15^ and likely on to the brainstem and diencephalic areas for further processing of nociceptive signals^4^. The majority of lamina I projection neurons and some in the deeper laminae express the NK1 receptor^59,60^, particularly those with a multipolar or fusiform shape^61^, and it is the latter which are Y1R-expressing^15^. However, consistent with previous studies^21^, we found that NPY-saporin did not change NK1R-immunoreactivity in the superficial dorsal horn, indicating that the mechanism by which NPY-saporin decreases neuropathic pain does not directly implicate NK1 receptor-expressing projection neurons. Furthermore, we do not believe that NPY-saporin targeted NK1 receptor-negative, large pyramidal-shaped projection neurons in lamina I^62^, because those do not express the Y1R receptor^15^. Although we cannot exclude a contribution of NK1 receptor-negative neurons in deeper laminae, their numbers are small and so we conclude that the mechanism by which NPY-saporin reduces neuropathic pain is most likely due to ablation of Y1R-expressing interneurons, rather than projection neurons, in the dorsal horn.

### The Y1R retains its functional responsiveness to the pain inhibitory actions of NPY in the setting of nerve injury

Peripheral nerve injury decreases the expression of opioid receptors^34,35^ and the ability of agonists to inhibit synaptic transmission in the dorsal horn^35^. Such mechanisms might explain the poor efficacy of opioid analgesics for neuropathic pain^36^. By contrast, we report that nerve injury did not decrease spinal Y1R expression. Instead, intrathecal administration of NPY reduced nerve injury-induced mechanical hyperalgesia as well as stimulus-evoked gene expression (using Fos as a marker) on the Y1R-expressing population of dorsal horn neurons, consistent with and extending our previous studies^9^. This supports our suggestion that the Y1R, in contrast to the mu opioid receptor, has greater capacity for endogenous pain relief^12^, and thus may be superior to opioids as a pharmacological target for long-lasting relief from neuropathic pain, particularly when administered at the spinal level^22^. In summary, our neuroanatomical and behavioral characterization of Y1R-expressing excitatory interneurons provides compelling evidence for the development of spinally-directed Y1R agonists to reduce chronic neuropathic pain.

### Does endogenous NPY act at Y1R-expressing neurons to tonically inhibit neuropathic pain?

When administered at the spinal level, NPY Y1 receptor agonists exert a broad-spectrum inhibition of pain^22^. This action is particularly robust in peripheral nerve injury models of neuropathic pain^11^, as intrathecal NPY has little effect on thermal thresholds in uninjured animals, but dose-dependently reduces behavioral signs of tactile and thermal hyperalgesia after injury, effects that can be blocked with a Y1R-selective antagonist^9^. These pharmacological actions mimic the tonic inhibitory control of neuropathic pain by endogenous NPY^12^. Indeed, our results are consistent with the hypothesis that NPY-Y1R signaling counters facilitatory mechanisms of neuropathic pain following peripheral nerve damage.

The classic Gate Control Theory (GCT) of pain postulated that the input generated by nociceptive as well as non-nociceptive afferents is regulated by a complex, gated circuit in the dorsal horn. One of the central tenets of GCT is that an inhibitory interneuron in the substantia gelatinosa responds to non-nociceptive input by inhibiting, or closing the gate on, a neuron that transmits pain messages to the brain^63^. Recent studies indicate that gating might also occur at excitatory interneurons, including those that express somatostatin, VGLUT3, or PKCγ^5,7,64^. Based on a large body of anatomical, behavioral, transcriptomic, and electrophysiological evidence, we speculate that the Y1R-expressing excitatory interneurons described here would be gated by inhibitory NPY-expressing interneurons that release NPY^22^. First, extensive anatomical evidence describes a large subset of GABA-expressing dorsal horn interneurons that co-express NPY^45^. Second, we reported that endogenous NPY tonically inhibits neuropathic pain behavior^12^. Third, Smith and colleagues described inhibitory actions of NPY on the neurophysiological activity of dorsal horn neurons^65^.

These results shed further light on the mechanism by which endogenous NPY tonically inhibits peripheral neuropathic pain, and we conclude this likely occurs via the hyperpolarization of Y1R-expressing excitatory interneurons, rather than through disinhibition of Y1R expressing inhibitory interneurons as we have earlier postulated^22^. Our results highlight the importance of endogenous NPY-Y1R signaling in chronic pain regulation and provide a foundational mechanism for the targeting of spinal Y1R-expressing excitatory interneurons as a promising target for pharmacotherapies to treat clinical neuropathic pain.

## Data Availability

The datasets generated during and/or analyzed during the current study will be made available from the corresponding author on request.
